# Supplementary material for: EnzML: multi-label prediction of enzyme classes using InterPro signatures
Source: BMC Bioinformatics. 2012 Apr 25;13:61. doi: 10.1186/1471-2105-13-61 (PMC3483700; doi:10.1186/1471-2105-13-61)
Supplement: Addtional file 5 — The Java code to format the data files, evaluate and predict. The file enzml_java_code.tar.gz contains the Java code used to format database data to ARFF and XML formats, to execute cross and train-test (jackknife) evaluations and to record evaluation results to database. More information is included in the readme.txt file and the Javadoc files. The code can be used with a MySQL database. To use a different database software, other JDBC drivers might be required. [file 1471-2105-13-61-S5.gz › java_code/utils/doc/uk/ac/ed/inf/utils/webutils/simpledomparser/XmlSearcher.html]

XmlSearcher


---


|  |  |  |
| --- | --- | --- |
| **Overview** \| **Package** \| **Class** \| **Use** \| **Tree** \| **Deprecated** \| **Index** \| **Help** | |  |
| **PREV CLASS**   NEXT CLASS | **FRAMES**    **NO FRAMES**     **All Classes** |  |
| SUMMARY: NESTED \| FIELD \| CONSTR \| METHOD | DETAIL: FIELD \| CONSTR \| METHOD |  |


---


## uk.ac.ed.inf.utils.webutils.simpledomparser Class XmlSearcher

```
java.lang.Object
  uk.ac.ed.inf.utils.webutils.simpledomparser.XmlSearcher
```

---

``` public class XmlSearcher extends java.lang.Object ```

Executes a search over a SimpleElement (an XML tree)

**Version:**
:   27 Feb 2009

**Author:**
:   Luna De Ferrari luna.deferrari-at-ed.ac.uk

---

| **Field Summary** | |
| --- | --- |
| `static boolean` | `m_debug` |


**Constructor Summary**

`XmlSearcher()`


| **Method Summary** | |
| --- | --- |
| `static XmlNode` | `depthFirstSearch(XmlNode xmlTree, java.lang.String searchString, java.lang.String attributeName, java.io.File log)`             Searches this tree in a depth first mode and returns the first node whose tag, text or attribute value matches the given string. |
| `static XmlNode` | `depthFirstSearch(XmlNode xmlTree, java.lang.String searchString, java.lang.String attributeName, java.io.File logFile, int emergencyBrake, int maxNumberOfChildren)`             Searches this tree in a depth first mode and returns the first node whose tag, text or attribute value matches the given string. |
| `static XmlNode` | `depthFirstSearchOfParent(XmlNode tree, java.lang.String desiredTag, java.lang.String attributeName)` |
| `static XmlNode` | `depthFirstSearchOfParent(XmlNode xmlTree, java.lang.String searchString, java.lang.String attributeName, java.io.File logFile, int emergencyBrake, int maxNumberOfChildren)`             TODO duplicated code! depthFirstSearch ~ depthFirstSearchOfParent Searches this tree in a depth first mode and returns the \*parent\* of the first node whose tag, text or attribute value matches the given string. |
| `static boolean` | `evaluateCondition(XmlNode element, java.lang.String wantedString)`             Check whether the desired string is either a tag or text for this element. |
| `static boolean` | `evaluateCondition(XmlNode element, java.lang.String wantedString, java.lang.String attributeName)`             Check whether the desired string is either a tag, text or an attribute \*value\* for this element. |
| `static java.util.LinkedList<XmlNode>` | `getInternalBlocks(java.lang.String externalBlockTag, XmlNode xmlTree, java.io.File log)`             Gets the children of a given xml tag |
| `static java.lang.String` | `getTextFromChildElement(XmlNode xmlTree, java.lang.String searchString, java.lang.String attributeName, java.io.File log)`             Checks if child exists and returns its text |

**Methods inherited from class java.lang.Object**

`equals, getClass, hashCode, notify, notifyAll, toString, wait, wait, wait`

**Field Detail**

### m\_debug

```
public static boolean m_debug
```


**Constructor Detail**

### XmlSearcher

```
public XmlSearcher()
```


**Method Detail**

### depthFirstSearch

```
public static XmlNode depthFirstSearch(XmlNode xmlTree,
                                       java.lang.String searchString,
                                       java.lang.String attributeName,
                                       java.io.File log)
```

:   Searches this tree in a depth first mode and returns the first node whose
    tag, text or attribute value matches the given string. If not found,
    stops after a default number of actions (usually 5000, but check the java
    code) or a certain number of children (usually 5000).

    :   **Parameters:**: `searchString` - the text to be found: `attributeName` - the attribute name (if available, but it can be null) **Returns:**: the first element whose tag/text/attribute value matches the wanted text

---


### depthFirstSearch

```
public static XmlNode depthFirstSearch(XmlNode xmlTree,
                                       java.lang.String searchString,
                                       java.lang.String attributeName,
                                       java.io.File logFile,
                                       int emergencyBrake,
                                       int maxNumberOfChildren)
```

:   Searches this tree in a depth first mode and returns the first node whose
    tag, text or attribute value matches the given string.

    :   **Parameters:**: `xmlTree` - the tree to be searched: `searchString` - the text to be found in a node: `attributeName` - the attribute name (if available, but it can be null): `logFile` - a log file (if a file output for the log is desired, or null to obtain a console printout): `emergencyBrake` - the number of tags to check before abandoning the search and returning null: `maxNumberOfChildren` - if a tag has more than this number of children, the children will not be considered in the search **Returns:**: the first element whose tag/text/attribute value matches the wanted text

---


### depthFirstSearchOfParent

```
public static XmlNode depthFirstSearchOfParent(XmlNode tree,
                                               java.lang.String desiredTag,
                                               java.lang.String attributeName)
```

---


### depthFirstSearchOfParent

```
public static XmlNode depthFirstSearchOfParent(XmlNode xmlTree,
                                               java.lang.String searchString,
                                               java.lang.String attributeName,
                                               java.io.File logFile,
                                               int emergencyBrake,
                                               int maxNumberOfChildren)
```

:   TODO duplicated code! depthFirstSearch ~ depthFirstSearchOfParent
    Searches this tree in a depth first mode and returns the \*parent\* of the
    first node whose tag, text or attribute value matches the given string.
    Having the parent allows, for example, to delete the child node.

    :   **Parameters:**: `xmlTree` - the tree to be searched: `searchString` - the text to be found in a node: `attributeName` - the attribute name (if available, but it can be null): `logFile` - a log file (if a file output for the log is desired, or null to obtain a console printout): `emergencyBrake` - the number of tags to check before abandoning the search and returning null: `maxNumberOfChildren` - if a tag has more than this number of children, the children will not be considered in the search **Returns:**: the \*parent\* of the first node whose tag/text/attribute value matches the wanted text

---


### evaluateCondition

```
public static boolean evaluateCondition(XmlNode element,
                                        java.lang.String wantedString)
```

:   Check whether the desired string is either a tag or text for this
    element.

    :   **Parameters:**: `element` -: `wantedString` - **Returns:**: the element if it matches, null otherwise

---


### evaluateCondition

```
public static boolean evaluateCondition(XmlNode element,
                                        java.lang.String wantedString,
                                        java.lang.String attributeName)
```

:   Check whether the desired string is either a tag, text or an attribute
    \*value\* for this element.

    :   **Parameters:**: `element` -: `wantedString` -: `attributeName` - if the attribute name is specified, the method checks the wanted string against the attribute value **Returns:**: the element if it matches, null otherwise

---


### getInternalBlocks

```
public static java.util.LinkedList<XmlNode> getInternalBlocks(java.lang.String externalBlockTag,
                                                              XmlNode xmlTree,
                                                              java.io.File log)
```

:   Gets the children of a given xml tag

    :   **Parameters:**: `externalBlockTag` - the tag that identifies the block **Returns:**: the children blocks insied the given xml tag

---


### getTextFromChildElement

```
public static java.lang.String getTextFromChildElement(XmlNode xmlTree,
                                                       java.lang.String searchString,
                                                       java.lang.String attributeName,
                                                       java.io.File log)
```

:   Checks if child exists and returns its text

    :   **Parameters:**: `searchString` - the tag or content string of the desired child: `attributeName` - the attribute name (if available, but it can be null if a search string is passed) **Returns:**: the text of the child or null if a child containing the search string could not be found


---


|  |  |  |
| --- | --- | --- |
| **Overview** \| **Package** \| **Class** \| **Use** \| **Tree** \| **Deprecated** \| **Index** \| **Help** | |  |
| **PREV CLASS**   NEXT CLASS | **FRAMES**    **NO FRAMES**     **All Classes** |  |
| SUMMARY: NESTED \| FIELD \| CONSTR \| METHOD | DETAIL: FIELD \| CONSTR \| METHOD |  |


---
